# A Tiered Approach to Exome Sequencing Analysis in Early-Onset Primary Ovarian Insufficiency

**DOI:** 10.1210/clinem/dgaf124

**Published:** 2025-02-25

**Authors:** Sinéad M McGlacken-Byrne, Jenifer P Suntharalingham, Miho Ishida, Federica Buonocore, Ignacio del Valle, Antoinette Cameron-Pimblett, Mehul T Dattani, John C Achermann, Gerard S Conway

**Affiliations:** Genetics and Genomic Medicine, UCL Great Ormond Street Institute of Child Health, University College London, London WC1N 1EH, UK; Institute for Women's Health, University College London, London WC1E 6AU, UK; Department of Paediatric Endocrinology, Great Ormond Street Hospital, London WC1N 3JH, UK; Genetics and Genomic Medicine, UCL Great Ormond Street Institute of Child Health, University College London, London WC1N 1EH, UK; Genetics and Genomic Medicine, UCL Great Ormond Street Institute of Child Health, University College London, London WC1N 1EH, UK; Genetics and Genomic Medicine, UCL Great Ormond Street Institute of Child Health, University College London, London WC1N 1EH, UK; Genetics and Genomic Medicine, UCL Great Ormond Street Institute of Child Health, University College London, London WC1N 1EH, UK; Institute for Women's Health, University College London, London WC1E 6AU, UK; Genetics and Genomic Medicine, UCL Great Ormond Street Institute of Child Health, University College London, London WC1N 1EH, UK; Department of Paediatric Endocrinology, Great Ormond Street Hospital, London WC1N 3JH, UK; Genetics and Genomic Medicine, UCL Great Ormond Street Institute of Child Health, University College London, London WC1N 1EH, UK; Institute for Women's Health, University College London, London WC1E 6AU, UK

**Keywords:** primary ovarian insufficiency, reproductive endocrinology, genetic analysis, primary amenorrhea, delayed puberty

## Abstract

**Context:**

Establishing the genetic basis of early-onset primary ovarian insufficiency (EO-POI, <25 years) is important, but defining variant pathogenicity is challenging.

**Objective:**

We aimed to elucidate the genetic architecture of EO-POI in a unique, large cohort. Young women with EO-POI (n = 149; n = 31 familial, n = 118 sporadic) attending a specialist reproductive unit were included. Exome sequencing was performed. After filtering, variants were retained that were: (1) rare/novel (minor allele frequency <0.01%); (2) predicted pathogenic/likely pathogenic; and (3) enriched in the cohort. Each variant was assigned to a category: Category 1, variants in Genomics England Primary Ovarian Insufficiency PanelApp genes (n = 69); Category 2, variants in other POI-associated genes (n = 355) or Category 1 variants following unexpected inheritance patterns; and Category 3, homozygous variants in novel candidate POI genes.

**Results:**

A total of 127 Category 1 or 2 variants were identified in 74 different genes (heterozygous 30.9%; homozygous 9.4%; polygenic 21.8%). In familial EO-POI, 64.7% (11/17 kindred) had a Category 1 or 2 variant identified (homozygous: *STAG3*, *MCM9*, *PSMC3IP*, *YTHDC2*, *ZSWIM7*; heterozygous: *POLR2C*, *NLRP11*, *IGSF10*, *PRKD1*, *PLEC*; polygenic: *PDE3A*, *POLR2H*, *MSH6*, *CLPP*). In sporadic EO-POI, 63.6% (n = 75/118) women had a variant identified: 21.2% (n = 25) Category 1; 42.4% (n = 50) Category 2. Novel POI candidate genes (Category 3) included *PCIF1*, *DND1*, *MEF2A*, *MMS22L*, *RXFP3*, *C4orf33*, and *ARRB1*.

**Conclusion:**

The genetic basis of EO-POI is complex and affected genes span ovarian developmental processes from fetal life to adulthood. Establishing the pathogenicity of individual heterozygous variants can be challenging. However, some women have clear monogenic causes, particularly in familial POI with autosomal recessive inheritance. Others have potential polygenic causes. We describe novel candidate POI genes warranting further exploration.

It is commonly accepted that the etiology of primary ovarian insufficiency (POI) is unknown for most women with the condition and this idiopathic group may have greater psychosocial sequelae (1, 2). A genetic basis for POI is evident, particularly in pedigrees with several affected members and in syndromic subgroups, and significant progress has been made in identifying causal pathogenic variants in recent years (3). In research contexts, a possible molecular genetic etiology has been proposed for up to 50% of women with a POI diagnosis (4-14). A genetic diagnosis in POI can not only provide an explanation for why they developed POI, but also facilitate individualized genetic counseling and tailored fertility preservation advice, allow for early identification of the condition in siblings of girls with familial POI, and alert clinicians to any features associated with POI (eg, hearing loss in POI associated with Perrault syndrome; increased cancer risk in certain variants within DNA repair genes) (15).

However, as genetic screening is more widely applied, there needs to be sufficient evidence supporting the pathogenicity of these variants to prevent poorly validated genetic variants being presented as confirmed genetic diagnoses to women with POI. Within the POI genetics field, the functional evidence for the pathogenicity of individual identified variants is highly variable between studies and often limited by small cohort size. Establishing causality has been further hindered by the complex genetic landscape of POI that has emerged to be remarkably heterogeneous. Variants in over 100 genes have now been associated with the pathogenesis of POI with multiple modes of inheritance proposed, including autosomal recessive, autosomal dominant, and oligogenic/polygenic. Taken together, this creates challenges when analyzing exome sequencing data in women with POI and deciding the extent to which variants are causative or contributory to the POI phenotype.

A further challenge in POI research is its variable phenotype. Most women with POI experience normal pubertal development and present later in adulthood with secondary amenorrhea, oligomenorrhea, or infertility. However, approximately 10% present with early-onset POI (EO-POI) characterized by primary amenorrhea or absent puberty (16, 17). POI in this age group represents the most severe end of the clinical spectrum, clearly far outside the normal physiological continuum for age at natural menopause (18). Given its severity, it may follow that young women with EO-POI are more likely to have a genetic diagnosis identified than older women with the same condition. Young women may also have qualitatively different underlying genetic mechanisms compared with those presenting at later ages. Preliminary evidence from the few studies that do separate out EO-POI within larger POI cohorts suggests that this may be the case: in 1 study, more pathogenic variants were found in women presenting with primary amenorrhea (25.8%) than in those presenting with secondary amenorrhea (17.8%), and a higher rate of biallelic variants was found in those with primary amenorrhea (5.8%) than in those with secondary amenorrhea (1.9%) (19). However, beyond these limited previous data, the clinical entity of EO-POI and its underlying genetic causes are not well understood.

Here, we aimed to characterize the genetic landscape of EO-POI using a tiered, objective, evidence-based approach. We performed exome sequencing in a large EO-POI cohort and developed a hierarchical approach to variant filtering based on different tiers of existing evidence for gene–disease relationships in POI. In doing so, we have elucidated important aspects of a complex genetic architecture underlying a particularly severe and often distressing subtype of POI.

## Materials and Methods

### Participant Recruitment

Women and girls with POI over the age of 16 years and their unaffected family members were invited to participate in the Reproductive Life Course Project (RLCP) at University College London Hospital (UCLH). All participants who joined the RLCP gave written, informed consent (if >18 years) and assent (if <18 years, along with cosigned parental consent) and continued to receive usual clinical care for POI at the Reproductive Medicine Unit of UCLH. The RLCP has ethical approval from the NRES Committee London-Chelsea (15/0877) and is sponsored by UCLH. Inclusion eligibility criteria included: (1) a confirmed POI diagnosis in accordance with the European Society of Human Reproduction and Embryology (ESHRE) guidelines (<40 years, amenorrhea >4 months, estrogen deficiency, and raised follicle-stimulating hormone >40 IU/L on 2 occasions at least 1 month apart) (20); (2) EO-POI (primary amenorrhea with or without absent puberty/pubertal arrest) and/or familial POI (2 or more first-degree or second-degree relatives within the same family with POI); (3) no underlying cause for POI identified (eg, iatrogenic POI); and (4) a 46,XX karyotype. All women underwent a Fragile X screen (*FMR1* analysis for CGG repeats). Women with a known genetic diagnosis of an established clinical syndrome definitively associated with POI were excluded (eg, confirmed Perrault syndrome). Otherwise, extra-ovarian clinical characteristics were recorded (Table 1). Unaffected family members of patients meeting the criteria above were also recruited with informed consent, if appropriate. The current and historic medical records at UCLH of all patients recruited to the RLCP were examined in detail. Data collected included demographic details, family history, details of diagnosis, current clinical status, and investigations performed to date.

**Table 1.** Clinical characteristics of the cohort

Clinical characteristic	Result
Fragile X screening	Negative (n = 149, 100%)
Karyotype	46,XX (n = 149, 100%)
Age at recruitment	28.3 years (IQR 7.2)
Ethnicity	Asian or Asian British—Indian 10.1% (n = 15)
	Asian or Asian British—Pakistani 11.4% (n = 17)
	Black or Black British—African 4.0% (n = 6)
	White British 59.7% (n = 89)
	White Irish 5.4% (n = 8)
	Other 9.4% (n = 14)
Weight	66.0 kg (IQR 14.8)
HRT	Yes 92.6% (n = 138)
	No 7.5% (n = 11)
FSH at diagnosis	95.0 IU/L (IQR 60.1)
LH at diagnosis	30.2 IU/L (IQR 18.5)
TPO antibodies	Positive 18.1% (n = 27)
	Negative 81.9% (n = 122)
Ovarian antibodies	Positive 0.7% (n = 1)
	Negative 99.3% (n = 148)
Adrenal antibodies	Positive 0.7% (n = 1)
	Negative 99.3% (n = 148)
Hypothyroidism on treatment	Yes 6.0% (n = 9)
	No 94.0% (n = 140)
Egg donation pregnancies	Yes 10.1% (n = 15)
	No 89.9% (n = 134)
Receiving psychological support	Yes 41.2% (n = 62)
	No 58.8% (n = 87)
Additional clinical features	Hyperparathyroidism (n = 1)
	Severe obesity undergoing bariatric surgery (n = 5)
	Congenital cataracts (n = 2)
	Growth hormone deficiency (n = 4)
	Uterus didelphys (n = 1)
	Congenital cardiac (n = 2)
	Myelodysplasia (n = 1)
	Type 1 diabetes mellitus (n = 1)
	Hemolytic anemia (n = 1)
	Autoimmune vitiligo (n = 1)
	Congenital deafness (n = 1)
	Retinal dystrophy (n = 1)
	Juvenile idiopathic arthritis (n = 1)

Abbreviations: FSH, follicle-stimulating hormone; HRT, hormone replacement therapy; LH, luteinizing hormone; TPO, thyroid peroxidase antibodies.

### DNA Collection and Extraction

Participants provided EDTA blood samples. DNA was extracted from whole blood using the QIAamp DNA Blood Midi/Maxi kit (QIAGEN N.V.) as per protocol.

### DNA Sequencing

An Exome CG enrichment panel (Nonacus, Birmingham, UK) was used for library preparation. Precapture library preparation and enrichment were performed following the Nonacus Library Prep v2 (HT) and Cell3 Target Enrichment protocol for Next Generation Sequencing protocols (Nonacus, Birmingham, UK). Processes were automated on the Hamilton StarLet robot (Hamilton, Reno. NV, USA). Library qualitative checks were undertaken using a Tapestation 4200 platform (Agilent, CA, USA). Libraries were sequenced on the NovaSeq 6000 (S4, 2 × 151 bp, Illumina) using paired-end sequencing and a S4 flowcell (UCL Genomics). Reads were aligned against the human reference genome sequence (NCBI, GRCh38) using the Burrows-Wheeler Aligner (BWA-MEM) (21). Platypus software (v0.8.1) was used for variant calling using standard parameters (22).

### Variant Analysis

Variant filtering was performed using QIAGEN Clinical Insight Interpret software (QIAGEN N.V. 2024). Variants with call quality ≥20 and a read depth ≥10 (median read depth 75; IQR 46-138) were kept. Unless the variant a well-established relationship with the pathogenesis of POI, filtering only retained variants that were (1) predicted pathogenic in silico on at least 2 of 3 pathogenicity prediction tools (Combined Annotation Dependent Depletion score >15 (23); PolyPhen2 (24); and SIFT (25)); (2) rare/novel (global minor allele frequency in gnomAD v4 <0.01% for biallelic variants (compound heterozygous/autosomal recessive); <0.005% for polygenic and single heterozygous variants; adjusted *P* < .0001); (3) enriched in the POI cohort compared with controls (gnomAD v4; 2-tailed Fisher exact testing); and (4) classified as pathogenic or likely pathogenic as per the American College of Medical Genetics (ACMG) criteria (26). All synonymous changes were excluded, unless they were associated with splice site loss up to 7 bases into an intron or predicted to affect splicing using MaxEntScan (27).

**Figure 1.**
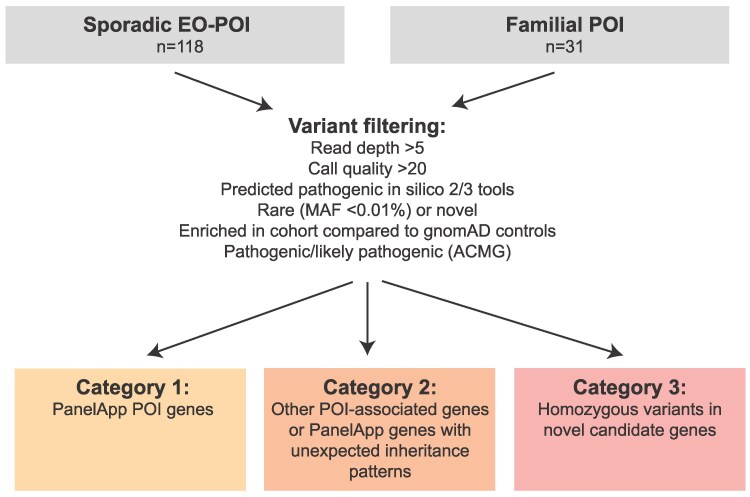
Variant filtering approach: Categories 1-3 were derived from the virtual POI gene panel used in the UK 100 000 genomes project on the genomics England primary ovarian insufficiency PanelApp (Jan 2024, v1.67; n = 69 genes; https://panelapp.genomicsengland.co.uk/panels/155/; Table S1 (28)); “Green” (high evidence for pathogenicity, n = 31 genes), “Amber” (emerging POI gene with moderate evidence, n = 22 genes), “Red” (emerging POI gene with low evidence, n = 16 genes) categories. POI, primary ovarian insufficiency; EO-POI, early-onset POI; MAF, minor allele frequency.

Downstream filtering then considered 3 categories of genes/variants. The entire cohort was initially screened for Category 1 variants. Category 1 variants included those within genes included on the Genomics England Primary Ovarian Insufficiency PanelApp (January 2024, v1.67; n = 69 genes; https://panelapp.genomicsengland.co.uk/panels/155/; Table S1 (28)), including those in the “Green” (n = 31 genes), “Amber” (n = 22 genes), and “Red” (n = 16 genes) categories. This is a virtual gene panel which was used in the UK 100 000 Genomes Project (a project where 100 000 genomes from 85 000 individuals accessing health care on the National Health Service [NHS] were sequenced), which uses expert crowdsourcing to assign genes associated with presumed monogenic phenotypes into 3 tiers (“Green”: high evidence for established pathogenicity in POI; “Amber”: emerging POI gene for which there is currently moderate evidence; “Red”: emerging POI gene for which there is currently low evidence). The entire cohort was then screened for a manually curated list of Category 2 variants (including in individuals carrying a Category 1 variant). These variants were those within genes associated with POI in published research studies not included on the POI PanelApp (n = 355 genes; supportive studies listed in Table S2 (28)) or variants in Category 1 genes included on the POI PanelApp but following different inheritance patterns to what is expected or established for that gene (eg, heterozygous variants in genes which usually follow recessive inheritance patterns, Table S1 (28)). Given that biallelic variants in key ovary-related genes are established causes of POI, the cohort was then screened for Category 3 variants, which were defined as homozygous variants in any gene meeting the above-described filtering criteria not included in Category 1 or 2, with the aim of identifying new candidate genes for POI (Fig. 1).

### Statistical Analysis

Statistical analysis (Fisher exact testing, 2-tailed) was performed using GraphPad Prism v9.1.1 (GraphPad Software). A *P* value of less than .05 was considered statistically significant. The Benjamini–Hochberg approach was used to adjust for multiple testing with cutoff adjusted *P* values of .05 (29).

## Results

### Clinical Characteristics of Study Population

**Figure 2.**
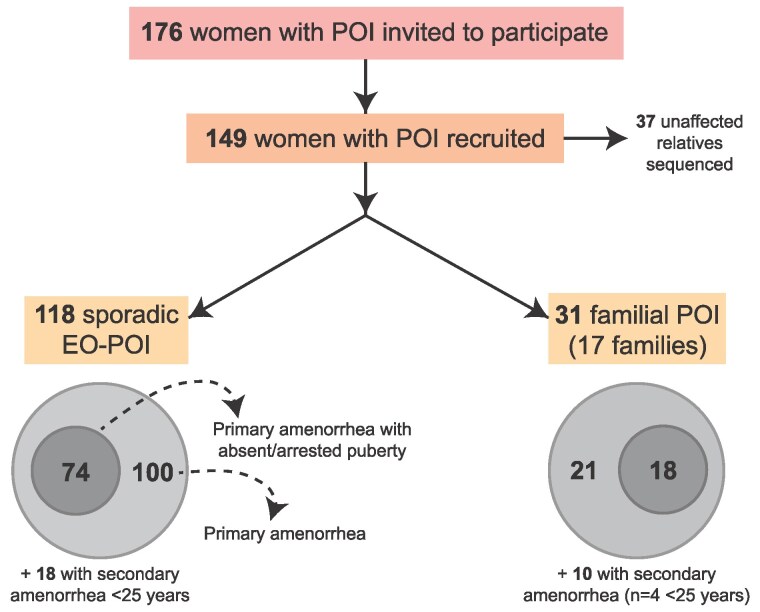
Flow diagram of POI exome study recruitment. A total of 199 with POI met the inclusion criteria, of which 23 were subsequently excluded due to known syndromic causes of POI (eg, galactosemia, Perrault syndrome). Of the remaining 176 women, 149 with POI were recruited. A further 37 unaffected relatives were recruited, meaning 186 individuals were sequenced in total. Participants were considered in 2 main groups: familial POI and sporadic EO-POI (POI <25 years). EO-POI, early-onset primary ovarian insufficiency.

A total of 199 women with EO-POI or familial POI attending UCLH met the inclusion criteria. Of these, 23 had syndromic POI (eg, Perrault syndrome, galactosemia) and were excluded. Of the remaining 176 women, a total of 149 women with POI were recruited to this study as part of the UCLH Reproductive Life Course Project (RLCP, Fig. 2) with informed consent. Of the entire cohort recruited (n = 149), 79.2% (n = 118) had sporadic, nonfamilial POI. The remainder of women, 20.8% (n = 31 individuals), had familial POI (ie, an affected sibling), coming from a total of 17 different kindreds of which 44.4% (n = 8) were consanguineous (Fig. 2). Of the total cohort, 96.0% (n = 143) had EO-POI as defined by onset of POI before the age of 25 years (the other 6 being siblings in familial POI kindred) and 81.2% (n = 121) presented with primary amenorrhea. Of those with primary amenorrhea, 76.0% (n = 92) presented with complete absence of puberty or premenarchal pubertal arrest; the remainder presented with early-onset secondary amenorrhea (POI presenting with absent menstrual cycles <25 years after a short period of normal cycling) or familial POI (Fig. 2). Unaffected family members were also recruited where possible (n = 37), with a focus on recruiting unaffected family members of women with familial POI (ie, 2 or more affected first- or second-degree family members with POI in the 1 kindred) (Table S3 (28)). However, parental DNA was not available in many cases.

Of the 118 women with sporadic EO-POI, 84.8% (n = 100) had primary amenorrhea and 62.7% (n = 74) had pubertal delay/arrest (Fig. 2). A small proportion (15.3%, n = 18) had transient menstrual cycles as young girls (mean age of menarche 11.5 years [IQR = 10.5-13.0]; mean age of last reproductive cycle 13 years [IQR = 15.5-20.5]). For those with familial POI, inclusion criteria were not restricted to EO-POI. However, most women within this group presented with primary amenorrhea (67.4%, n = 21) and for those secondary amenorrhea (32.3%, n = 10), mean age of menarche was 13.1 years (IQR = 12.1-14.9) and mean age of last menstrual period was 22.7 years (IQR = 16.0-27.0). Only 6 women with familial POI had non-EO-POI. Notably, across the entire cohort of 149 women, none had a positive *FMR1* premutation (Fragile X) screen and only 1 woman had positive ovarian and adrenal antibodies, despite all participants being tested (to note, as positive antibodies did not rule out a participant also having a genetic mechanism for POI identified, women with positive antibodies was still included for genetic analysis). Other clinical characteristics are outlined in Table 1.

### Genetic Findings in the Familial POI Cohort

**Figure 3.**
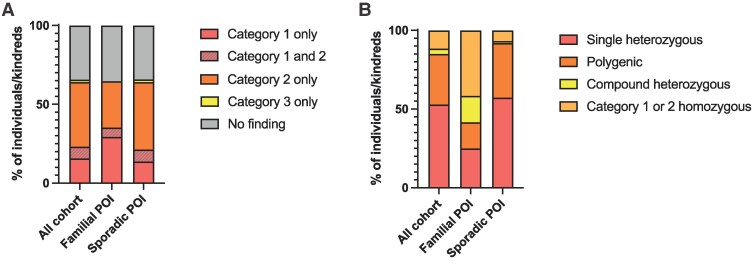
Variants identified in POI cohort. (A) Variant by category across the cohort. Proportion of individuals/kindred (women from the same kindred considered as 1 entity for the purpose of this figure) across the sporadic and familial POI cohort with Category 1, Category 2, and Category 3 variants or combinations thereof. (B) Variant by probable mode of inheritance across the cohort. Proportion of individuals/kindred across the sporadic and familial POI cohort with single rare heterozygous variants, homozygous variants, likely compound heterozygous variants (note: parental DNA was often not available to confirm variants in trans), and variants in 2 or more different genes (likely polygenic). Only Category 1 or 2 variants are shown here. Those individuals without any variant identified or those with Category 3 variants (candidate homozygous variants) are not included in this figure. POI, primary ovarian insufficiency.

Variant filtering followed processes outlined in “Materials and Methods” and Fig. 1 selecting only rare (<0.01% gnomAD 4.0)/novel variants that are predicted pathogenic in multiple in silico models. As described in “Materials and Methods,” variants were segregated into Category 1 (variant in PanelApp POI gene, n = 69); Category 2 (variant in gene associated with POI in the literature but not included on PanelApp, totaling 355 genes as listed in Table S2 (28)) (or, PanelApp variant following an unexpected inheritance pattern); and Category 3 (forward screen for homozygous variants in novel candidate POI gene). Of 17 kindred with familial POI, 11 kindred (64.7%), including 20 affected women, had a genetic variant identified in a Category 1 or 2 gene (Table 2 and Fig. 3A and 3B; Table S4 (28)). Of these 17 kindred, 6 kindred (35.3%) had variants in a Category 1 gene: homozygous variants in *STAG3*, *MCM9*, *PSMC3IP*, *YTHDC2*, and *ZSWIM7*; and a heterozygous variant in *POLR2C* (32) (note: the *YTHDC2* and *ZSWIM7* variants have been published separately (30, 31)) (Table 2). A further 5 families (29.4%) had Category 2 variant(s), including 3 kindred with single heterozygous variants in *NLRP11* (33), *IGSF10* (4), and *PRKD1* (4); another kindred with compound heterozygous variants in *PLEC* (34); and a further family with oligogenic variants in *PDE3A* (9), *POLR3H* (35), *MSH6* (36), and *CLPP* (37) (Table S4 (28)). Notably, the participant with the Category 1 *POLR2C* variant also had heterozygous variants within Category 2 genes. The remaining 6 families (35.3%) had no genetic finding identified. Only 1 of the 8 consanguineous pedigrees did not have a genetic finding identified.

**Table 2.** Variants detected in the familial primary ovarian insufficiency cohort

Kindred	Gene	Transcript	Genomic variant	Protein variant	Zygosity	dbSNP ID	Inheritance pattern	Population frequency
**Category 1 gene**								
PanelApp “Green”								
FPOI1*^a^*	*STAG3*	NM_001282717	c.2301 + 2T>G	NA	Biallelic	NA	AR	0.0000006195
FPOI2*^a^*	*MCM9*	NM_017696.3	c.1217C>T	p.A406V	Biallelic	NA	AR	0.00000062
	*XRCC1^b^*	NM_006297.3	c.482C>A	p.P161Q	Biallelic	NA		0.00000063
PanelApp “Amber”								
FPOI3*^a^*	*PSMC3IP*	NM_016556.4	c.35-2A>G	NA	Biallelic	NA	AR	0.00000000
FPOI4	*POLR2C*	NM_032940.3	c.670G>A	p.G224S	Monoallelic	NA	Polygenic	0.00001492
	*ANKRD^b^*	*NM_001372053.1*	*c.3641G>C*	*p.S1214T*	*Monoallelic*	775823393		0.00005914
	*PCSK1^b^*	*NM_000439.5*	*c.548delC*	*p.P183Qfs*	*Monoallelic*	NA		0.00000000
	*STAG3^b^*	*NM_001282717.2*	*c.1437_1446del*	*p.Q479Hfs^b^*	*Monoallelic*	*NA*		0.00000000
	*TP63^b^*	*NM_001329146.2*	*c.97G>A*	*p.G33S*	*Monoallelic*	*375508394*		0.00007249
	*WRN^b^*	*NM_000553.6*	*c.1957C>G*	*p.L653V*	*Monoallelic*	*373177461*		0.00000744
PanelApp “Red”								
FPOI5*^a^*	*ZSWIM7^c^*	NM_001042697.2	c.173C>G	p.S58*^b^*	Biallelic	NA	AR	0.00000000
FPOI6*^a^*	*YTHDC2^d^*	NM_022828.5	c.2567C>G	p.P856R	Biallelic	NA	AR	0.00000000
**Category 2 gene**								
FPOI7	*NLRP11*	NM_001394894.2	c.2318C>G	p.P773R	Monoallelic	NA	AD	0.00000186
FPOI8	*PLEC*	NM_201384.3	c.5933A>C	p.E1978A	Monoallelic	782017511	CH	0.00005263
	*PLEC*	NM_201384.3	c.-98T>G	NA	Monoallelic	1833681895		0.00001377
FPOI9	*IGSF10*	NM_178822.5	c.5996dup	p.S2000Ifs*^b^*	Monoallelic	770294032	AD	0.00015780
FPOI10	*PRKD1*	NM_002742.3	c.724G>T	p.G242C	Monoallelic	1042589965	AD	0.00028700
FPOI11	*PDE3A*	NM_000921.5	c.1210A>C	p.N404H	Monoallelic	765851273	Polygenic	0.00003942
	*POLR3H*	NM_001018050.4	c.445C>T	p.R149C	Monoallelic	NA		0.00005913
	*MSH6*	NM_000179.3	c.1508C>G	p.S503C	Monoallelic	63750897		0.00054560
	*CLPP*	NM_006012.4	c.411G>C	p.Q137H	Monoallelic	NA		0.00000434

*FPOI12*, *FPOI13*, *FPOI14*, *FPOI15*, *FPOI16*, *FPOI17* no finding identified.

Probable compound heterozygote; parental DNA not available for testing.

Population frequency: gnomAD 4.0 allele frequency.

Abbreviations: FPOI, familial POI; AR, homozygous variant, autosomal recessive inheritance; AD, heterozygous variant, autosomal dominant inheritance; CH, likely compound heterozygous inheritance.

^*a*^Consanguineous family.

^*b*^Category 2 gene.

^*c*^Previously published in McGlacken-Byrne et al (*JCEM*, 2022) (30).

^*d*^Previously published in McGlacken-Byrne et al (*JCI Insight*, 2022) (31).

### Genetic Findings in the Sporadic POI Cohort

Of women with sporadic POI, 21.2% (n = 25/118) had a Category 1 variant(s) (n = 8 homozygous; n = 3 with 2 different variants in same gene (possible compound heterozygous); n = 14 single heterozygous variants) (Table 3, Table S5 (28)). Genes included 12 green PanelApp genes; 4 orange PanelApp genes; and 4 red PanelApp genes. These 25 women were then screened for Category 2 variants, and 9 had 1 or more Category 2 variants in addition to the Category 1 variant(s), inferring possible oligogenic inheritance in these women (Fig. 3A; Table S5 (28)). The remaining cohort of women with sporadic POI were then screened for Category 2 variants, which were found in a further 42.4% (n = 50/118 further women; 16 digenic/polygenic; 34 heterozygous; none biallelic). When both Category 1 and 2 variants were considered, 75 women (63.6%) had at least 1 Category 1 or 2 variant. In total, Category 1 or 2 variants in 2 or more genes were identified in 26/118 women with sporadic POI (22.0%), suggesting possible oligogenic or polygenic inheritance in this proportion.

**Table 3.** Variants detected in the sporadic primary ovarian insufficiency cohort

ID	Gene	Transcript	Genomic variant	Protein variant	Zygosity	Inheritance pattern	Population frequency
**Category 1 gene**							
PanelApp “Green”							
SPOI1	*FSHR*	NM_000145.4	c.300-2A>G		Monoallelic	Oligogenic	0.00000000
	*FSHR*	NM_000145.4	c.1220C>A	p.A407D	Monoallelic		0.00000657
	*HELQ^a^*	NM_001297756.2	c.227G>A	p.S113N	Monoallelic		0.00005257
	*MLH1^a^*	NM_001258274.3	c.859C>T	p.R44C	Monoallelic		0.00004617
SPOI2	*FSHR*	NM_000145.4	c.1854C>A	p.N592K	Biallelic	AR	0.00000000
SPOI3	*GDF9*	NM_001288828.3	c.530T>G	p.L2658	Monoallelic	Oligogenic	0.00000657
	*GDF9*	NM_001288828.3	c.340C>T	p.Q114*^a^*	Monoallelic		0.00008540
	*PRDM9*	NM_020227.4	c.2306C>G	p.T769R	Monoallelic		0.00043609
SPOI4*^e^*	*MCM9*	NM_017696.3	c.724G>A	p.G242R	Biallelic	AR	0.00002630
SPOI5	*MCM9*	NM_001378363.1	c.905-1652G>T		Monoallelic	CH	0.00000658
	*MCM9*	NM_001378367.1	c.1523G>A	p.R532Q	Monoallelic		0.00000000
SPOI6	*PRDM9*	NM_020227.4	c.2233G>A	p.E745K	Monoallelic	AD	0.00140452
SPOI7	*PRDM9*	NM_020227.4	c.2306C>G	p.T769R	Monoallelic	AD	0.00043609
SPOI8	*BMP15*	NM_005448.2	c.911G>T	p.G304V	Monoallelic	AD	0.00001804
SPOI9	*NBN*	NM_002485.5	c.625C>T	p.Q291*^a^*	Biallelic	Oligogenic	0.00000399
	*ATG7^a^*	NM_001136031.3	c.1277C>T	p.P387L	Monoallelic		0.00092667
	*GREM1^a^*	NM_013372.7	c.525G>C	p.Q105H	Monoallelic		0.00000657
SPOI10	*BMP15*	NM_005448.2	c.443T>C	p.L148P	Monoallelic	Oligogenic	0.00764884
	*AMH^a^*	NM_000479.5	c.790G>C	p.G264R	Monoallelic		0.00042718
SPOI11	*GDF9*	NM_001288828.3	c.199A>C	p.K67Q	Monoallelic	Oligogenic	0.00001972
	*BRCA2^a^*	NM_000059.4	c.6231G>C	p.K2077N	Monoallelic		0.00004600
SPOI12	*BMP15*	NM_005448.2	c.443T>C	p.L148P	Monoallelic	AD	0.00764884
SPOI13	*NOBOX*	NM_001080413.3	c.1345C>T	p.R332*^a^*	Biallelic	Oligogenic	0.00001993
	*SETX^a^*	NM_001351527.2	c.1504C>T	p.R502W	Monoallelic		0.00028904
SPOI14	*BMP15*	NM_005448.2	c.443T>C	p.L148P	Monoallelic	Oligogenic	0.00764884
	*DCAF17^a^*	NM_001321157.22	c.61T>G	p.F21V	Monoallelic		0.00004602
	*FANCM^a^*	NM_020937.4	c.1478G>A	p.G519D	Monoallelic		0.00001315
SPOI15	*STAG3*	NM_001282717.2	c.942-3T>G		Biallelic	AR	0.00000000
SPOI16	*LARS2*	NM_015340.4	c.1565C>A	p.T522N	Biallelic	Oligogenic	0.00000000
	*MEIOB^a^*	NM_001163560.3	c.902A>G	p.Y301C	Monoallelic		0.00000000
	*PRDM1^a^*	NM_001198.4	c.239A>T	p.E44V	Monoallelic		0.00000000
SPOI17*^e^*	*MCM8*	NM_032485.6	c.698delC	p.P233fs*^a^*64	Biallelic	AR	0.00000000
PaneApp “Amber”							
SPOI18	*EIF4ENIF1*	NM_001164502.2	c.511C>T	p.R171C	Monoallelic	AD	0.00001591
	*PCIF1^b^*	NM_022104.4	c.830G>A	p.R277Q	Biallelic		0.00000800
SPOI19	*EIF4ENIF1*	NM_001164502.2	c.865C>G	p.Q289E	Monoallelic	AD	0.00055203
SPOI20	*BUB1B*	NM_001128628.3	c.2847C>G	p.I935M	Monoallelic	AD	0.00007887
SPOI21	*GDF9*	NM_014402.5	c.145G>T	p.V49L	Monoallelic	Oligogenic	0.00000000
	*SALL4^a^*	NM_020436.5	c.1070A>G	p.K357R	Monoallelic		0.00007889
PanelApp “Red”							
SPOI22	*NBN*	NM_002485.5	c.-259-2A>G		Monoallelic	AD	0.00000399
SPOI23	*FOXO4*	NM_002015.4	c.1016C>T	p.P394L	Monoallelic	Oligogenic	0.00001740
	*GJA4^a^*	NM_002060.3	c.98G>A	p.R33H	Monoallelic		0.00003903
	*MMS22L^b^*	NM_001350599.2	c.92G>T	p.C31F	Biallelic		0.00004601
** SPOI24**	***ATM***	NM_001351834.2	c.1837G>T	p.V613L	Monoallelic	AD	0.00003946
** SPOI25*^c,e^***	***YTHDC2***	NM_022828.5	c.1129G>T	p.E377*^a^*	Biallelic	AR	0.00000000
**Category 2 gene**							
SPOI26	*FSHR*	NM_000145.4	c.1639C>T	p.R547C	Monoallelic	Oligogenic	0.00000399
	*MLH1*	NM_001258274.3	c.326delC	p.P317fs*^a^*17	Monoallelic		
SPOI27	*FANCF*	NM_022725.4	c.385C>G	p.L129V	Monoallelic	Oligogenic	0.00047291
	*MCM8*	NM_032485.6	c.72C>G	p.D24E	Monoallelic		
SPOI28*^c^*	*BMP8B*	NM_001195007.2	c.1198G>A	p.G400S	Monoallelic	Oligogenic	0.00040727
	*SETX*	NM_001351527.2	c.1282T>A	p.Y428N	Monoallelic		0.00000000
	*PKD1L1^b^*	NM_138295.5	c.111G>A	p.W37^a^	Biallelic		0.00000000
	*DND1^b^*	NM_194249.3	c.917G>A	p.W306^a^	Biallelic		0.00000000
SPOI29	*ZNF462*	NM_021224.6	c.629A>G	p.E1365G	Monoallelic	AD	0.00009858
SPOI30	*MLH1*	NM_001258274.3	c.859C>T	p.R44C	Monoallelic	AD	0.00004617
SPOI31	*GHR*	NM_001242460.1	c.660G>T	p.L227F	Monoallelic	AD	0.00005257
SPOI32	*BMPR2*	NM_001204.7	c.1042G>A	p.V348I	Monoallelic	Oligogenic	0.00024317
	*BNC1*	NM_001717.4	c.70C>T	p.R24W	Monoallelic		0.00009425
	*MEF2A^b^*	NM_001319206.4	c.293G>C	p.S98T	Biallelic		0.00000000
SPOI33	*MLH1*	NM_001354622.2	c.478A>T	p.N160Y	Monoallelic	AD	0.00000657
SPOI34	*FANCL*	NM_001130480.2	n.977_980dupATTA	p.T367fs*^a^*	Monoallelic	Oligogenic	0.00552348
	*MCM9*	NM_001378363.1	c.713A>G	p.N238S	Monoallelic		0.00342412
SPOI35	*BRCA2*	NM_000059.4	c.10045A>G	p.T3349A	Monoallelic	Oligogenic	0.00009199
	*SYCP1*	NM_001282542.2	c.2425G>A	p.D809N	Monoallelic		0.00015797
SPOI36	*LLGL1*	NM_004140.4	c.1982G>A	p.R661H	Monoallelic	AD	0.00008539
SPOI37	*NRIP1*	NM_003489.4	c.1997T>C	p.I666T	Monoallelic	AD	0.00000657
	*RXFP3^b^*	NM_016568.3	c.1381G>A	p.D461N	Biallelic		0.00000000
SPOI38	*ESR1*	NM_001385568.1	c.1514G>A	p.R242Q	Monoallelic	Oligogenic	0.00000000
	*INSL3*	NM_005543.4	c.145C>T	p.P49S	Monoallelic		0.00007226
SPOI39	*SETX*	NM_001351527.2	c.654G>C	p.K218N	Monoallelic	AD	0.00037464
SPOI40	*BMPR2*	NM_001204.7	c.2104A>C	p.T702P	Monoallelic	AD	0.00000372
SPOI41	*BRCA2*	NM_000059.4	c.9038C>T	p.T3013I	Monoallelic	AD	0.00021685
SPOI42	*PATL2*	NM_001387261.1	c.541G>A	p.G181R	Monoallelic	AD	0.00000660
SPOI43	*PATL2*	NM_001387261.1	c.466C>T	p.P156S	Monoallelic	Oligogenic	0.00059805
	*SUN1*	NM_001367694.1	c.1361T>C	p.F486S	Monoallelic		0.00000000
SPOI44	*HELQ*	NM_001297756.2	c.2552A>G	p.Y307C	Monoallelic	AD	0.00019660
SPOI45	*BMPR2*	NM_001204.7	c.2140G>T	p.A714S	Monoallelic	Oligogenic	0.00028923
	*LHX8*	NM_001001933.1	c.974C>T	p.A315V	Monoallelic		0.00148545
	*RNF212*	NR_159498.1	c.170A>T	p.H57L	Monoallelic		0.00012480
SPOI46	*STAG3*	NM_001282717.2	c.423C>A	p.C141*^a^*	Monoallelic	Oligogenic	0.00003287
	*SUN1*	NM_001367694.1	n.472C>T	p.R91C	Monoallelic		0.00008542
SPOI47	*PATL2*	NM_001387261.1	c.86A>G	p.E29G	Monoallelic	AD	0.00078927
SPOI48	*MEIOB*	NM_001163560.3	c.814C>T	p.R272*^a^*	Monoallelic	AD	0.00003290
SPOI49	*AMH*	NM_000479.5	c.995A>G	p.D332G	Monoallelic	Oligogenic	0.00001318
	*PATL2*	NM_001387261.1	c.469C>T	p.R157W	Monoallelic		0.00001971
SPOI50	*BLM*	NM_001287247.2	c.3878A>G	p.E918G	Monoallelic	Oligogenic	0.00002628
	*MEI1*	NM_152513.4	c.1346G>C	p.S449T	Monoallelic		0.00000186
SPOI51	*FANCM*	NM_020937.4	c.504G>C	p.M168I	Monoallelic	Oligogenic	0.00020375
	*ZP1*	NM_001391943.1	c.461A>T	p.D49V	Monoallelic		0.00000000
SPOI52	*STAG3*	NM_001282717.2	c.466T>C	p.S156P	Monoallelic	AD	0.00091328
SPOI53	*ANKRD31*	NM_001164443.1	c.1804C>T	p.R602C	Monoallelic	AD	0.00001316
SPOI54	*IRS4*	NM_001379150.1	c.1889C>T	p.P630L	Monoallelic	AD	0.00000083
SPOI55	*ESR1*	NM_001385568.1	c.811C>T	p.R268C	Monoallelic	AD	0.00036183
SPOI56	*FSHB*	NM_001382289.1	c.254C>T	p.A85V	Monoallelic	AD	0.00004599
SPOI57	*MACF2*	NM_001144769.5	c.109T>C	p.C37R	Monoallelic	AD	0.00028915
SPOI58	*SMC1B*	NM_001291501.2	c.175A>G	p.K59E	Monoallelic	AD	0.00017742
SPOI59	*SETX*	NM_001351527.2	c.5591A>C	p.Q1864P	Monoallelic	AD	0.00003943
SPOI60	*AMH*	NM_000479.5	c.635_651del	p.L212fs*^a^*165	Monoallelic	AD	0.00000000
SPOI61	*ZNF462*	NM_021224.6	c.400C>G	p.P1289A	Monoallelic	AD	0.00003287
SPOI62	*DMC1*	NM_001363017.2	c.385C>T	p.R129C	Monoallelic	AD	0.00010520
SPOI63	*TSC2*	NM_001318829.2	c.4910G>A	p.R1591H	Monoallelic	AD	0.00007227
SPOI64	*FANCL*	NM_018062.4	c.784A>G	p.M262V	Monoallelic	Oligogenic	0.00028959
	*UBR2*	NM_001363705.2	c.4290G>T	p.Q1430H	Monoallelic		0.00000000
SPOI65	*BRCA2*	NM_000059.4	c.8614G>A	p.E2872K	Monoallelic	Oligogenic	0.00000186
	*SETX*	NM_001351527.2	c.3281A>G	p.Q1094R	Monoallelic		0.00013795
	*UBR2*	NM_001363705.2	c.1259A>G	p.Q420R	Monoallelic		0.00005916
SPOI66	*BMPR2*	NM_001204.7	c.2948G>A	p.R983Q	Monoallelic	AD	0.00030896
SPOI67	*FIGNL1*	NM_022116.7	c.1186_1190	p.S507fs*^a^*5	Monoallelic	AD	0.00036204
SPOI68	*EXO1*	NM_006027.4	c.797C>G	p.T266R	Monoallelic	AD	0.00002140
SPOI69	*GHR*	NM_001242460.1	c.620G>A	p.R207H	Monoallelic	AD	0.00080836
SPOI70	*TSC2*	NM_001318829.2	c.274C>T	p.R100C	Monoallelic	AD	0.00089400
SPOI71	*MCM9*	NM_001378363.1	c.713A>G	p.N238S	Monoallelic	AD	0.00342412
SPOI72	*TSC2*	NM_001318829.2	c.3788C>A	p.S1392Y	Monoallelic	AD	0.00003285
SPOI73	*KIT*	NM_001385292.1	c.2683C>G	p.P896A	Monoallelic	Oligogenic	0.00002045
	*REC8*	NM_005132.3	c.329T>C	p.M110T	Monoallelic		0.00060451
	*ZNF462*	NM_021224.6	c.509A>G	p.K170R	Monoallelic		0.00000657
SPOI74	*MLH3*	NM_014381.3	c.2711C>A	p.S904Y	Monoallelic	AD	0.00000658
SPOI75	*MCM9*	NM_001378356.1	c.1689 + 589C>G	p.L639V	Monoallelic	AD	0.00084118
**Category 3 gene**							
SPOI76	*ARRB1*	NM_004041.5	c.709C>T	p.Q237*^a^*	Biallelic	AR	0.00000000
SPOI77*^d,e^*	*C4ORF33*	NM_001099783.2	c.182T>C	p.V61A	Biallelic	AR	0.00000659
	*ABCA4*	NM_000350.3	c.6729 + 5_6729 + 19del	p.F2161Cfs*^a^*3	Biallelic	AR	0.00001315

Population frequency: gnomAD 4.0 allele frequency.

SPOI78-118 had no finding identified.

Probable compound heterozygote; parental DNA not available for testing.

Abbreviations: AR, homozygous variant, autosomal recessive inheritance; AD, heterozygous variant, autosomal dominant inheritance; CH, likely compound heterozygous inheritance; SPOI, sporadic primary ovarian insufficiency.

^*a*^Category 2 gene.

^*b*^Category 3 gene.

^*c*^Previously published in McGlacken-Byrne et al (JCI Insight, 2022 (31)).

^*d*^SPOI27: POI and situs inversus; SPOI77: POI and rod–cone retinal dystrophy; SPOI78: POI and chronic fatigue.

^*e*^Consanguineous family.

### Novel Candidate POI Genes

To identify novel genetic causes of POI, the cohort was then screened for Category 3 homozygous variants. Category 3 variants possibly associated with the POI phenotype in existing model systems/animal models were identified in 7 women (Table 4; Table S5 (28)). Of the 75 women with Category 1 or 2 variants, 5 women had 1 or more Category 3 variants (in *PCIF1*, *MMS22L*, *DND1*, *MEF2A*, *RXFP3*). Of the remainder without Category 1 or 2 variants, a further 2 women had 1 or more Category 3 variants potentially explaining their phenotype in 2 POI candidates *(C4ORF33* and *ARRB1*). Homozygous variants in *PDK1L1* and *ABCA4* were also identified in 2 women (the woman with the *DND1* variant and the *C4orf33* variant, respectively), but in these cases these variants likely explained other aspects of the participant's clinical presentation rather than POI as both variants have been definitively associated with these other phenotypes (situs inversus and rod–cone retinal dystrophy, respectively).

**Table 4.** Proposed function and supportive evidence for identified variants

Kindred	Gene	Genomic change	Protein change	Known function	Supportive evidence for gonadal phenotype
SPOI19	*PCIF1*	c.830G>A	p.R277Q	N6,2-O-dimethyladenosine methyltransferase	Pcif1 mutant flies have reduced fertility (particularly females)
SPOI24	*MMS22L*	c.92G>T	p.C31F	DNA repair	Required for meiotic homologous recombination with RAD51
SPOI28	*DND1*	c.917G>A	p.W306*	Inhibits microRNA-mediated repression	DND1-dependent mRNA destabilization required for primordial germ cell survival in mice; DND1 homozygous variants associated with male non-obstructive azoospermia
SPOI32	*MEF2A*	c.293G>C	p.S98T	Myogenic functions	Required for normal germ cell development in mouse follicular cells (female knockouts only partially fertile)
SPOI37	*RXFP3*	c.1381G>A	p.D461N	DNA damage response	Expressed during folliculogenesis in mice
SPOI76	*ARRB1*	c.709C>T	p.Q237*	Hippo signaling v via YAP interaction	YAP1 expression required for primordial follicle growth
SPOI77	*C4ORF33*	c.182T>C	p.V61A	Unknown	Highly expressed in the testis and epididymis in the Human Protein Atlas

Abbreviation: SPOI, sporadic primary ovarian insufficiency.

### POI Has a Heterogenous Genetic Landscape

**Figure 4.**
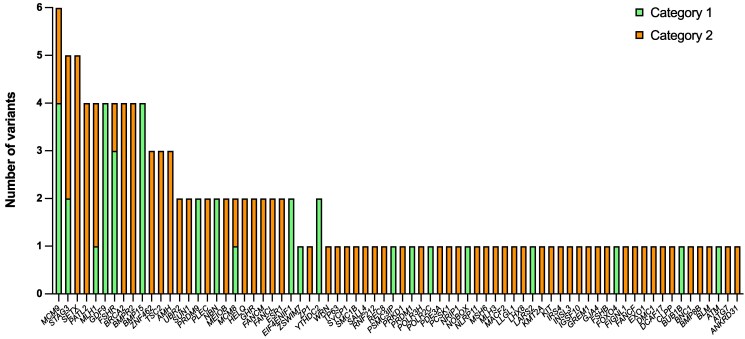
Category 1 and 2 variants across the cohort. The 74 genes in which Category 1 or 2 variants (n = 128) were identified in this study cohort are shown (x-axis). The number of different variants in each identified POI gene are shown (y-axis). Individual variants are represented once in the graph, even if present in 2 or more individuals in the cohort. Compound heterozygous variants are included as 2 different variants (n = 2). Homozygous variants are included once (n = 1).

Taking Category 1 and 2 variants together across both familial and sporadic cohorts, a total of 128 variants in 74 different genes previously associated with POI were identified in 63.1% (n = 95) women in the cohort studied (11 kindreds composed of 20 recruited individuals with familial POI, and 75 individuals with sporadic POI, as described above) (Figs. 3A and 4; Table S6 (28)). Considering women from the same kindred as 1 entity, a total of 86 individual women/kindred across the combined familial/sporadic cohort had Category 1 or 2 variants identified: likely oligogenic/polygenic variants in 32.2% (n = 28/86); homozygous variants in 11.5% (n = 10/86); presumed compound heterozygous variants in 3.4% (n = 3/86; parents not available for testing for these individuals, so variants could not be proven in trans); and single rare heterozygous variants in 52.9% (n = 46/86) (Fig. 3B). Notably, of these 46 heterozygous variants, although individually rare (<0.01% minor allele frequency [MAF]), 42 (91.3%) are also found in the heterozygote state in normal population controls (gnomAD 4.0) (Tables S4 and S5 (28)). In addition to Category 1 and/or 2 variants, a total of 11 women carried homozygous variants in 13 candidate genes not previously associated with POI, including 6 with variants also identified from Category 1 or 2 genes (Fig. 3B; Tables S4 and 5 (28)).

Results were then compared between the familial and sporadic POI groups. More homozygous variants were found in familial POI than in sporadic (35.3% [n = 6/17] compared with 4.2% [n = 5/118]) (Fisher exact test, *P* < .001) and a nonstatistically significant trend towards more heterozygous variants found in sporadic POI compared with familial (36.4% [n = 43] compared with 17.6% [n = 3]) (Fig. 3A). Additionally, more Category 1 variants were found in the familial cohort than in the sporadic cohort (29.4% [5/17] compared with 13.6% [16/118]) (Fig. 3B).

**Figure 5.**
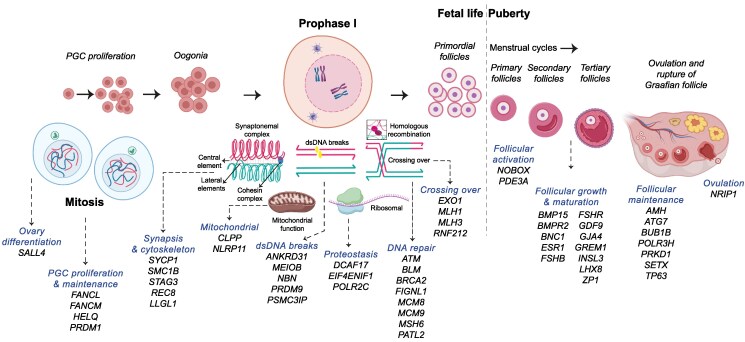
Mapping POI-associated genes to key ovarian biological processes over a reproductive life course. POI-associated genes in which variants were identified in this cohort are mapped to key individual biological and cellular processes in fetal and adult ovarian development. PGC, primordial germ cell; dsDNA, double-stranded DNA. This figure was created in part on BioRender.com. Transformative figure; from McGlacken-Byrne et al JCEM (30). © The authors.

Proposed biological functions of identified genes as they relate to ovarian function (if known), as well as selected studies in which other variants in these genes have been described in POI, are listed in Table S7 (28). These processes span an entire reproductive life course and included ovary differentiation and development; primordial germ cell migration and growth, oogonia proliferation, meiosis (particularly prophase I in fetal life); folliculogenesis and maturation; maintenance of the resting follicle pool; and, ultimately, ovulation to produce an oocyte capable of fertilization (Fig. 5). Four or more different variants were described in *STAG3*, *SETX*, *PATL2*, *MLH1*, *MCM9*, *GDF9*, *FSHR*, *BRCA2*, *BMPR2*, and *BMP15* (Fig. 4). For most genes, only 1 variant within it was described. Most variants described were missense variants (90.7%, n = 117/128) with a small proportion (9.3%, n = 12/128) frameshift or stop gain loss of function variants. Of the 11 women carrying homozygous Category 3 variants in new POI candidate genes, 3 were loss of function variants (stop gain).

## Discussion

Here, we describe rare genetic variants in POI-associated genes in a high proportion of women with EO-POI and/or familial POI. The real-life cohort presented is mixed ancestry and uniquely enriched for early-onset, nonsyndromic POI; to our knowledge, it is the largest cohort of EO-POI described to date (19). Additionally, the very high recruitment rate reduces the risk of negative selection bias that might have confounded results. Our “detection rate” is overall similar to those reported by other exome sequencing studies over the last 5 years, many of which also identified variants in POI-associated genes in over half of the POI cohort examined (5, 6, 9, 13). This adds weight to the emerging concept that a greater proportion of POI has a genetic basis than previously thought. We specifically advance our understanding of the genetic landscape of EO-POI. Importantly too, our judicious, evidence-based approach to exome sequencing analysis acknowledges that association does not necessarily imply causality. Key insights and reasons for caution are outlined below.

Firstly, our data provide insight into the contribution of heterozygous variants to the POI phenotype. In both familial and sporadic POI, we found a high proportion of both rare/novel, predicted deleterious single heterozygous and digenic/oligogenic variants in genes from a carefully curated gene list based on previously proposed POI genes. Our variant identification rate is similar to or higher than previous studies sequencing mixed POI cohorts (5, 6, 9, 13). Although our study focused on EO-POI, 1 large, published cohort described an EO-POI subset, and, similar to our “Category 1” detection rate in our data, found that 25.8% of women within the EO-POI group had a pathogenic or likely pathogenic variant in a POI-associated gene. Taken together, these studies suggest that at least a proportion of POI is oligogenic in origin. At face value, the high proportion of women with POI carrying rare heterozygote variants in POI-associated genes also suggests an inheritance model whereby haploinsufficiency of key POI-associated genes gives rise to clinical POI. However, this enrichment of rare heterozygous variants in POI-associated genes within POI cohorts needs to be considered alongside the presence or absence of these variants in population controls; the exponential rise in the number of genes associated with POI in recent years; and the fact that the strength of evidence underlying these individual gene–disease relationships significantly varies in the first instance. Some gene–disease relationships in POI are well-established, having been replicated in several cohorts and/or supported by functional evidence and convincing animal models. Many of these well-established genes are included on the “Green” Genomics England PanelApp (eg, *MCM9* and *STAG3*) (38, 39). Other gene–disease relationships in POI are less well established, having been described in only single cases or small cohorts with limited or no supporting functional evidence linking proposed loss of function variants within these genes to POI. Furthermore, in recent years, variants in several POI genes that classically follow recessive modes of inheritance (eg, *MCM8*, *BRCA2*, *MCM9*) have been reported in POI cohorts in heterozygous states, sometimes without convincing evidence for the contribution of haploinsufficiency to the POI phenotype (23).

With these issues in mind, we used a hierarchical and categorical approach to variant filtering in this study which clearly highlighted the different tiers of evidence supporting gene–disease relationships in POI and allowed results to be divided into higher evidence (Category 1) and lower evidence (Category 2 and 3). The step of searching for individual heterozygous variants in population controls also revealed that most single heterozygous variants identified, although individually rare (MAF < 0.01%), were also found in presumed healthy gnomAD 4.0 controls. This echoes findings from a recent paper which evaluated the penetrance of heterozygous variants in POI-associated genes in both POI and control cohorts and found limited evidence for the pathogenicity of these variants in a heterozygote state (40). This does not mean that these identified variants have no possible relationship to POI but does call into question whether these single heterozygous variants can give rise to a POI phenotype in isolation. It may be that carrying multiple heterozygous variants in different genes contributes to a POI phenotype in a complex trait rather than Mendelian inheritance pattern (and that our stringent filtering approach missed other potential variants of significance in our cohort). The high rate of polygenicity in POI-associated genes suggested by this study would support this concept.

Secondly, we propose that a clearly defined subgroup of women have autosomal recessively inherited—truly “monogenic”—POI. We identified a significant proportion of women—approximately 10%—carrying *homozygous* variants in Category 1 genes in our cohort, many of which have established pathogenicity in POI when inherited in an autosomal recessive manner (eg, *MCM8*, *MCM9*, *STAG3*, and *PSMC3IP*). Unsurprisingly, consanguineous kindred had a high incidence of autosomal recessive POI; all but 1 had a homozygous genetic variant identified. This figure of 10% is higher than previously reported studies, including those with EO-POI mixed within their cohorts (19, 33). This suggests that women with EO-POI are more likely to carry recessive variants in POI associated genes than those with later-onset POI. Many of these genes have clearly defined functions in human ovarian development and function (eg, DNA repair, meiosis). It may be that recessively inherited, EO-POI represents 1 end of a genotype–phenotype spectrum, with POI as complex polygenic trait more commonly defining POI occurring at a later age. Potentially, carrying an increasing number of these polygenic pathogenic variants could translate to a more severe POI phenotype. For a further group of women, POI may represent a complex trait phenotype lying just outside the continuum of normal age at natural, nonpathological menopause reflecting a burden of common single-nucleotide polymorphisms associated with earlier menopausal age. It is possible too that certain common single-nucleotide polymorphisms may confer genetic vulnerability to gene–environment effects that increase an individual risk of developing POI or physiological menopause at an earlier than expected age. Ultimately, larger and POI-specific genome-wide association studies and exome-wide rare variant association studies will be required to definitively answer these questions.

Thirdly, we advance our understanding of recessively mediated POI by proposing some convincing novel candidate genes for POI worthy of further exploration. Our gene-agnostic approach of searching for recessively inherited variants across the cohort resulted in the identification of promising novel candidate genes for POI. Indeed, the evidence for some of these candidate homozygous variants is possibly stronger than the evidence underpinning some of heterozygous variants described in Category 2 POI-associated genes in this study. We discuss these novel candidate genes below.

A subset of candidate genes for POI identified in this study—including *PCIF1*, *DND1*, and *MEF2A—*have convincing corresponding animal models of gonadal insufficiency and therefore warrant further investigation in humans. A homozygous variant in *PCIF1*, a methyltransferase of N6,2-O-dimethyladenosine (41), found in a woman with sporadic POI adds weight to the emerging view that complex epitranscriptomic regulatory networks are key for normal ovary development (42). Furthermore, *Pcif1* mutant flies have a reduced fertility phenotype that is exaggerated in females (43). *DND1* homozygous variants have been identified in males with nonobstructive azoospermia (44) and a novel stop–gain homozygous variant was identified here in a woman with sporadic POI and situs inversus from a consanguineous family. Mouse models have demonstrated that DND1-dependent mRNA destabilization is required for primordial germ cell survival in mice (45, 46); we suggest a role for it in female ovarian function in humans. Notably, this woman also had a homozygous variant in *PKD1L1* which likely independently explains the situs inversus phenotype (47, 48). We also identified a recessive variant in *MEF2A*, a gene with established myogenic functions, suggesting a possible role for it in human female ovarian function. *Mef2* has been shown to have a role in normal germ cell development in mouse follicular cells, with *Mef2* females only partially fertile (49). Specifically, it has been proposed to regulate expression of the nuclear receptor *Nr4a1* in mice (50).

Pathogenic variants in genes involved in DNA repair (including *BRCA2*, *ZSWIM7*, *MCM9)* are recognized causes of POI, and we propose 2 further DNA repair candidates here. Firstly, a homozygous variant was found in *MMS22L*, which together with *RAD51* is required for functioning homologous recombination. *RAD51* has been previously implicated in the pathogenesis of POI (36, 51); *MMS22L* represents a plausible new POI candidate gene. *RXFP3* is another DNA repair gene known to regulate the DNA damage response pathway via its ligand, relaxin 3 (52). Several relaxin genes, including *RXFP3*, are expressed in male reproductive organs and during folliculogenesis in mice (53, 54); it may therefore have an unexplored role in human gonadal function.

The remaining 2 homozygous Category 3 variants identified in this study, *C4orf33* and *ARRB1*, do not have a recognized role in gonadal development but warrant further exploration. The woman with the *C4orf33* variant is from a consanguineous family and has both POI and rod–cone retinal dystrophy with 2 separate homozygous variants identified: a missense variant in *C4orf33* and a frameshift variant in *ABCA4.* The latter is an established cause of rod–cone dystrophy and likely explains her ophthalmological phenotype (55). This highlights that, while some “nonsyndromic” genetically mediated POI is associated with other features arising from the same genetic variant (eg, hypopituitarism and POI resulting from pathogenic *RNPC3* variants) (56), in some women—particularly from consanguineous families—there can be 2 *separate* recessive variants explaining complex phenotypes. The other variant identified in this woman, *C4orf33*, is highly expressed in the testis and epididymis in males in the Human Protein Atlas but has no known function in the ovary. The remaining Category 3 gene identified in this study, *ARRB1* (encoding B-arrestin), functions in mice as part of the Hippo signaling pathway, interacting with YAP to modulate downstream transcription of YAP responsive genes (57). *YAP1* expression is required for normal human ovarian development, important for primordial follicle growth and activation (58). Additionally, the NF2–YAP pathway has been associated with POI previously, with variants in *BNC1* resulting in an ovarian insufficiency phenotype (59).

Taken together, our work advances our understanding of the genetic architecture of POI. We identify several avenues for further exploration as well as reasons for caution. Like others, we found genetic variants in genes previously associated with POI in a high proportion of this POI cohort. While this does support the concept that a significant fraction of POI has an underlying genetic contribution, we caution that a genetic finding does not equal a genetic diagnosis. Specifically, the strength of evidence supporting the pathogenicity of each identified variant, and the evidence for its proposed model of inheritance, needs to be considered. For those variants with limited functional evidence (here named Category 2), we suggest there is a need to revisit gene–disease relationships in the first instance. We also propose that a proportion of POI is polygenic in origin, possibly constituting the severe end of a complex trait phenotypic spectrum of age at natural menopause. We also conclude that a distinct subset of EO-POI is truly monogenic, particularly in the context of consanguinity, and arises frequently from homozygous variants in key, established POI-associated genes. These genes frequently have functions related to pivotal ovary developmental pathways such as meiosis and DNA repair; indeed, pathogenic variants within these genes were among the first identified in sequencing studies of consanguineous POI pedigrees in the early days of genetic testing for POI. The complexity and uncertainty around genetic testing in POI has clinical consequences: for instance, beyond a recommendation to perform a karyotype and Fragile X screen, there is no publicly available targeted gene panel for POI in the UK (ie, as part of the NHS National Genomic Test Directory). Clearly, there is a pressing need to elucidate the genetic architecture of POI more fully to guide which variants should be routinely returned to women with POI as a genetic diagnosis and which genes should (or should not) be included on panels available for routine clinical genetic testing in the future. At the same time, exploring in more detail the novel candidate genes identified in this study would accelerate our understanding of human ovarian function and, in time, may provide a convincing genetic diagnosis to a greater proportion of women with POI.

## Data Availability

Restrictions apply to the availability of some or all data generated or analyzed during this study to preserve patient confidentiality. The corresponding author will on request detail the restrictions and any conditions under which access to some data may be provided.

## Abbreviations

EO-POI: early-onset primary ovarian insufficiency
MAF: minor allele frequency
RLCP: Reproductive Life Course Project
UCLH: University College London Hospital
